# A retrospective analysis of pathogen distribution and antimicrobial resistance trends of bacteria in a tertiary hospital in China, 2021–2025

**DOI:** 10.3389/fcimb.2026.1856155

**Published:** 2026-07-15

**Authors:** Zihuan Li, Maorui Lin, Huarun Li, Tian Wang, Guanwen Lin, Ting Fan, Ya Zou

**Affiliations:** 1 Department of Infection Prevention and Control, The Affiliated Guangdong Second Provincial General Hospital of Jinan University, Guangzhou, Guangdong, China; 2 Department of Laboratory Medicine, The Affiliated Guangdong Second Provincial General Hospital of Jinan University, Guangzhou, Guangdong, China; 3 Department of Dermatology, The Affiliated Guangdong Second Provincial General Hospital of Jinan University, Guangzhou, Guangdong, China

**Keywords:** antimicrobial resistance, gram-negative bacteria, infection prevention and control, multidrug-resistant organisms, pathogen distribution

## Abstract

**Background:**

Antimicrobial resistance (AMR) poses a significant global health threat, with multidrug-resistant organisms (MDROs) increasingly contributing to healthcare-associated infections (HAIs). Understanding local pathogen distribution and resistance trends is essential for guiding empirical therapy and infection control strategies. This study aims to analyze the pathogen distribution and AMR trends of bacteria in a tertiary hospital in South China from 2021 to 2025.

**Methods:**

A retrospective analysis was conducted on bacterial isolates collected from a tertiary hospital in Guangzhou, China, from 2021 to 2025. Bacterial identification and antimicrobial susceptibility testing were performed using the VITEK 2 automated system and the Kirby-Bauer disk diffusion method, with results interpreted according to CLSI guidelines. Annual data analysis was conducted using WHONET 5.6 software. Antimicrobial consumption was expressed as defined daily doses (DDD) per 1,000 patient-days, and incidence of HAIs caused by MDROs was calculated as cases per 1,000 patient-days.

**Results:**

A total of 25,326 non-duplicate bacterial isolates were analyzed. Gram-negative bacteria predominated throughout the study period, with *Escherichia coli*, *Klebsiella pneumoniae*, *Pseudomonas aeruginosa*, and *Acinetobacter baumannii* being the most frequently isolated species. Among Gram-positive bacteria, *Staphylococcus aureus* showed a marked increase from 2023 onward. *Escherichia coli* exhibited persistently high resistance to fluoroquinolones (>57%) and third-generation cephalosporins (>50%), with an increasing trend in carbapenem resistance. *Klebsiella pneumoniae* demonstrated declining resistance to carbapenems but a concerning emergence of tigecycline resistance in 2025 (6.2%). *Acinetobacter baumannii* maintained high carbapenem resistance (>60%) throughout, with colistin and tigecycline remaining the only consistently effective agents (resistance ≤1.9% and ≤1.8%, respectively). Antimicrobial consumption showed a sustained increase in carbapenem use (from 21.78 to 47.06 DDD per 1,000 patient-days), while incidence of HAIs caused by MDROs declined from 0.30 to 0.12 cases per 1,000 patient-days following intensified infection prevention and control measures implemented in 2024.

**Conclusions:**

This study reveals a persistently high burden of MDROs in a South China tertiary hospital, particularly among Gram-negative pathogens. Despite increasing antimicrobial consumption, enhanced IPC measures, including active surveillance and improved contact isolation compliance, effectively reduced HAIs-MDROs incidence. These findings underscore the critical importance of integrated antimicrobial stewardship and infection control strategies in combating AMR.

## Introduction

The emergence and spread of antimicrobial resistance (AMR) not only poses a grave threat to global health by steadily undermining our capacity to prevent and treat a broadening range of bacterial infections but also stands as one of the most urgent public health challenges of the 21st century (Antimicrobial resistance: a top ten global public health threat, 2021; Sati et al., 2025; Martinez-Melendez et al., 2026). Multidrug-resistant organisms (MDROs) are increasingly responsible for healthcare-associated infections (HAIs), contributing to elevated morbidity and mortality, prolonged hospital stays, and escalating healthcare costs (Naylor et al., 2019; Global burden of bacterial antimicrobial resistance in 2019: a systematic analysis, 2022; Ajulo and Awosile, 2024; Global burden of bacterial antimicrobial resistance 1990-2021: a systematic analysis with forecasts to 2050, 2024; Zha et al., 2025).

The World Health Organization has categorized carbapenem-resistant *Acinetobacter baumannii* (CRAB), along with *Enterobacterales* that are resistant to both carbapenems and third-generation cephalosporins, as the highest-priority pathogens. Additionally, vancomycin-resistant *Enterococcus faecium* (VRE), carbapenem-resistant *Pseudomonas aeruginosa* (CRPA), and methicillin-resistant *Staphylococcus aureus* (MRSA) are classified as high-priority pathogens (Sati et al., 2025).

Over the years, the introduction and expanded use of new antimicrobials have driven the emergence and spread of multidrug-resistant bacterial pathogens. Nevertheless, pathogen fitness (Fuzi, 2025), clonal expansion (Lawal et al., 2022; Ikhimiukor et al., 2024; Wang et al., 2024), and transmission (McCarthy et al., 2025) are equally critical drivers that must be considered for a comprehensive understanding of antimicrobial resistance dynamics. This has contributed to a rise in HAI caused by key MDROs, including *Escherichia coli* (*E. coli*), *Acinetobacter baumannii* (*A. baumannii*), *Klebsiella pneumoniae* (*K. pneumoniae*), and *Staphylococcus aureus* (*S. aureus*) (Miller and Arias, 2024; Hetta et al., 2025).

In China, high detection rates have been observed for CRAB, followed by MRSA, CRPA, and carbapenem-resistant *Klebsiella pneumoniae* (CRKP). Among these, CRAB and CRKP represent the most prominent MDROs associated with HAI in the country (Zong et al., 2020). The continued upward trend in the prevalence of MDROs underscores the growing severity of AMR among clinically significant pathogens and remains a pressing public health concern that warrants sustained surveillance and intervention efforts (Yao et al., 2025). The pathogenic bacteria identified in East China were chiefly occupied by *E. coli*, *K. pneumoniae* and *Pseudomonas aeruginosa* (*P. aeruginosa*) *(*Liu L. et al., 2025).

The primary surveillance systems responsible for monitoring bacterial resistance at the national level in China include the China Antimicrobial Resistance Surveillance System (CARSS) and the China Antimicrobial Surveillance Network (CHINET) (Qin et al., 2024). They track the dissemination of bacterial pathogens and resistance profiles across major referral hospitals (Hu et al., 2020).

Investigating the distribution patterns and AMR trends of bacterial pathogens in hospitals located in South China represents a critical research priority. However, published data focusing on this region remain limited. Our institution, a large-scale tertiary general hospital in South China, integrates medical care, education, scientific research, and health management, and serves as a major referral center in the region. To combat the rising threat of MDROs, our hospital implemented a series of enhanced infection prevention and control measures in 2024. Key components included weekly active surveillance for MDROs combined with immediate notification, on-site audit, and timely feedback to ensure prompt contact isolation. These measures have been previously described (Li et al., 2026a).

This study aims to address the regional surveillance gap by providing comprehensive AMR data from our hospital. As a representative tertiary teaching hospital in South China, our findings are intended to inform regional antimicrobial stewardship programs. We compiled and analyzed bacterial surveillance data collected at our institution from 2021 to 2025, examining pathogen distribution, susceptibility profiles, and temporal trends in AMR patterns. Ultimately, this study seeks to provide evidence-based references to support hospital departments in formulating antimicrobial use policies and optimizing clinical application.

## Materials and methods

### Setting

This retrospective study investigated the pathogen distribution and antimicrobial resistance trends of bacteria in the Affiliated Guangdong Second Provincial General Hospital of Jinan University. This tertiary care institution in Guangzhou, South China, has a capacity of approximately 1,730 beds.

### Bacteria identification and antimicrobial susceptibility testing

Duplicate isolates from the same anatomical site and patient were removed. Specimens were collected under standard aseptic conditions. All bacterial isolates were identified using the VITEK 2 automated system. Antimicrobial susceptibility testing was performed in accordance with the guidelines established by the Clinical and Laboratory Standards Institute (CLSI). Susceptibility profiles were determined using both the automated system and the Kirby–Bauer (KB) disk diffusion method. Results were interpreted based on CLSI breakpoints, and annual data analysis was conducted using WHONET 5.6 software. WHONET is a windows-based software designed to manage and analyze microbiology laboratory data, with a special focus on antimicrobial susceptibility test results (Aboushady et al., 2026). In this study, WHONET 5.6 software was used to calculate annual resistance rates and generate summary tables of antimicrobial susceptibility data for each bacterial species.

### Antimicrobial consumption and healthcare-associated MDROs infections from 2021 to 2025

This study analyzed the annual consumption of carbapenems (ertapenem, meropenem, and imipenem/cilastatin), cephalosporins, fluoroquinolones, and glycopeptides from 2021 to 2025, expressed as defined daily doses (DDD) per 1,000 patient-days. The incidence of healthcare-associated MDROs infections, including MRSA, carbapenem-resistant *Escherichia coli* (CREC), CRKP, CRPA, CRAB, and VRE, was expressed as cases per 1,000 patient-days. Each case of healthcare-associated MDROs infection was confirmed jointly by infection control specialists and clinical physicians.

### analyse Statistical s

The constituent ratio of each bacterial species was defined as the number of isolates of that species detected in a given year divided by the total number of all bacterial isolates detected in the same year, expressed as a percentage. The annual resistance rate of a given bacterial species to a specific antimicrobial agent was calculated as the number of resistant isolates divided by the total number of isolates of that bacterial species tested in the same year, expressed as a percentage. The annual detection rate of a given MDROs was calculated as the number of MDROs isolates divided by the total number of isolates of the corresponding pathogen tested in the same year, expressed as a percentage. The antimicrobial consumption data of selected classes of antimicrobial agents were analyzed by year and expressed as the defined daily dose (DDD) per 1,000 patient days. The annual incidence rate of HAIs caused by a specific MDROs was calculated as the number of new events due to that MDROs divided by the cumulative patient-days during the same period and expressed as cases per 1,000 patient-days. If a patient had more than one episode of a HAI event due to the same pathogen with the same hospital number, only the first episode was recorded, regardless of the specimen source. All statistical analyses were performed using IBM SPSS statistical software (version 29.0). All statistical analyses were evaluated at the statistical significance level of *p* < 0.05 (two-sided).

## Results

### Distribution of specimen types from 2021 to 2025

**Table 1** presents the annual quantities of various positive specimens identified from 2021 to 2025. The total number of positive specimens peaked in 2023 at 5,584 before declining slightly in the following years. Sputum consistently remained the most frequently detected specimen type, while urine specimens showed steady growth throughout the period.

**Table 1:** Distribution of specimen types from 2021 to 2025.

Specimen type	2021	2022	2023	2024	2025
Sputum	1286	1130	1534	1311	1120
Urine	1018	1133	1309	1222	1259
Blood	539	536	606	680	515
Wound swab	393	346	434	441	509
Pus	237	193	222	299	204
Bronchoalveolar lavage fluid	209	271	366	287	363
Semen	135	89	182	117	125
Secretion	109	93	144	156	157
Puncture fluid	55	54	72	72	91
Tissue	20	15	25	54	70
Other specimen types	786	665	690	743	635
Total	4787	4525	5584	5382	5048

### Distribution and antimicrobial resistance of gram-positive and gram-negative bacteria

**Figure 1:**
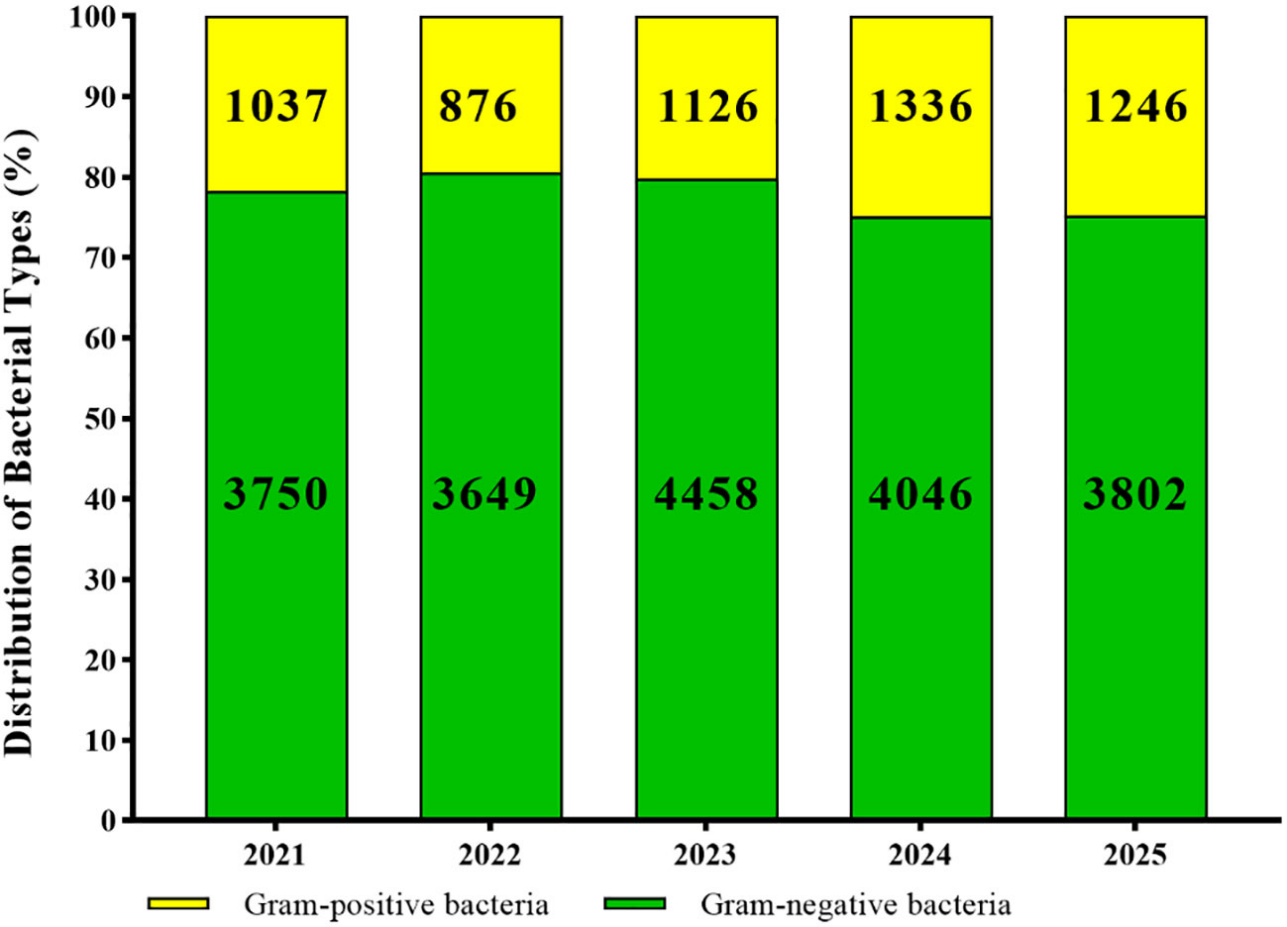
Distribution of bacteria types.

**Figure 1** shows the distribution of bacterial types, indicating an increasing trend in the proportion of Gram-positive bacteria after 2024. As shown in **Tables 2**, **3**, the annual resistant proportion of Gram-positive and Gram-negative bacteria to various antimicrobial agents exhibited dynamic changes between 2021 and 2025.

**Table 2:** Annual resistant proportion of gram-positive bacteria.

Antibiotic	2021 (n = 1037)	2022 (n = 876)	2023 (n = 1126)	2024 (n = 1336)	2025 (n = 1246)
Ampicillin	78.0	82.0	90.2	89.0	89.0
Penicillin G	77.5	82.8	90.1	89.1	89.1
Erythromycin	55.9	54.5	58.0	56.4	52.3
Oxacillin	49.6	56.1	52.0	46.4	44.0
Clindamycin	24.8	32.6	24.7	28.8	28.9
Levofloxacin	36.7	39.1	38.6	36.7	35.1
Moxifloxacin	22.8	25.9	24.7	22.1	22.0
Gentamicin	15.4	17.6	17.2	15.6	15.5
Trimethoprim/Sulfamethoxazole	16.7	11.3	18.2	11.0	12.1
Rifampin	6.0	4.2	4.1	5.2	3.9
Quinupristin/Dalfopristin	0.0	1.0	0.0	0.0	0.0
Ceftaroline	0.0	0.0	0.0	0.0	0.0
Tigecycline	0.0	0.0	0.0	0.0	0.0
Teicoplanin	0.0	0.0	1.0	1.5	2.9
Vancomycin	0.0	0.0	1.0	1.3	2.5
Linezolid	0.0	0.0	0.0	0.0	0.0

**Table 3:** Annual resistant proportion of gram-negative bacteria.

Antibiotic	2021 (n = 3750)	2022 (n = 3649)	2023 (n = 4458)	2024 (n = 4046)	2025 (n = 3802)
Cefuroxime	52.3	53.0	50.0	49.8	47.8
Ceftriaxone	51.3	51.6	49.3	47.7	45.3
Trimethoprim/Sulfamethoxazole	45.3	45.5	45.9	38.6	38.5
Ciprofloxacin	45.1	46.5	41.1	39.3	33.9
Levofloxacin	43.4	44.5	40.8	39.3	36.7
Aztreonam	22.1	27.3	25.8	29.8	27.7
Cefepime	38.7	31.3	28.4	26.7	25.9
Ceftazidime	30.3	32.2	27.9	25.6	25.4
Ticarcillin/Clavulanate	29.7	31.3	28.9	29.2	28.9
Amoxicillin/Clavulanate	26.1	27.3	25.4	20.6	17.4
Tobramycin	19.2	22.0	20.4	16.9	24.9
Cefoxitin	24.5	27.6	24.3	23.5	17.8
Piperacillin/Tazobactam	23.5	25.2	26.8	23.9	23.4
Meropenem	23.5	25.1	23.1	23.0	24.1
Imipenem	19.2	20.9	19.7	17.6	16.6
Cefoperazone/Sulbactam	19.4	21.8	18.9	15.4	13.9
Chloramphenicol	10.9	12.6	5.1	7.7	22.4
Amikacin	8.0	9.6	9.1	7.9	9.1
Colistin	6.2	3.6	8.2	4.4	3.0
Ertapenem	11.4	14.3	12.5	10.6	9.1
Tigecycline	6.0	4.8	6.2	11.6	2.5

Annual resistant proportion among Gram-positive bacteria to agents such as ampicillin and penicillin G remained consistently high (>77.5%). In contrast, while Gram-negative bacteria showed generally high resistance to third-generation cephalosporins (e.g., ceftriaxone, cefuroxime) and fluoroquinolones (e.g., ciprofloxacin), most of these proportion demonstrated a gradual declining trend.

Notably, a slight but observable increase in resistance to teicoplanin and vancomycin among Gram-positive bacteria emerged from 2023 onward (rising from 1.0% to 2.9% and from 1.0% to 2.5%, respectively), warranting attention.

Gram-positive bacteria maintained negligible or very low resistance (0% or ≤1.0%) to quinupristin/dalfopristin, ceftaroline, tigecycline, and linezolid throughout the five-year period, indicating preserved potent activity of these antimicrobials. Similarly, resistance to rifampin remained low (3.9%-6.0%).

Among Gram-negative bacteria, annual resistant proportion to amikacin (7.9%-9.6%), colistin (3.0%-8.2%), and imipenem (16.6%-20.9%) were sustained at relatively low overall levels.

### Distribution of pathogen types from 2021 to 2025

The epidemiological landscape from 2021 to 2025 was dominated by Gram-negative pathogens, notably *E. coli*, *K. pneumoniae*, *P. aeruginosa*, and *A. baumannii*. Among Gram-positive bacteria, *S. aureus* emerged as the predominant pathogen, with a significant increase in isolates observed from 2023 onward. **Table 4** illustrates the distribution of pathogen types from 2021 to 2025.

**Table 4:** Distribution of pathogen types from 2021 to 2025.

Pathogen	2021	2022	2023	2024	2025
Gram-positive bacteria					
Staphylococcus aureus	397	319	363	503	488
Enterococcus faecalis	179	152	197	192	176
Staphylococcus epidermidis	101	73	81	80	56
Enterococcus faecium	85	72	102	135	110
Streptococcus agalactiae	61	44	122	110	119
Others Gram-Positive Bacteria	214	216	261	316	297
Gram-negative bacteria					
Escherichia coli	1059	1096	1325	1239	1229
Klebsiella pneumoniae	853	877	1059	832	746
Pseudomonas aeruginosa	563	518	662	592	603
Acinetobacter baumannii	410	339	454	334	264
Proteus penneri	127	105	144	114	55
Others Gram-Negative Bacteria	738	714	814	935	905
Total	4787	4525	5584	5382	5048

### Antimicrobial resistance trends in common bacterial pathogens

**Tables 5**–**8** summarize the antimicrobial annual resistant proportion for five major bacterial pathogens from 2021 to 2025: *S. aureus*, *E. coli*, *K. pneumoniae*, *P. aeruginosa*, and *A. baumannii*.

**Table 5:** Annual resistant proportion of *Escherichia coli* from 2021 to 2025.

Antibiotic	2021 (n = 1059)	2022 (n = 1096)	2023 (n = 1325)	2024 (n = 1239)	2025 (n = 1229)
Cefuroxime	53.1	52.0	50.7	52.7	50.5
Ceftriaxone	54.4	52.8	51.1	53.5	50.2
Trimethoprim/Sulfamethoxazole	55.2	52.3	53.4	48.4	47.0
Ciprofloxacin	63.5	57.4	62.5	67.1	63.3
Levofloxacin	52.7	50.1	50.4	53.3	51.3
Aztreonam	24.6	25.1	25.3	28.3	34.7
Cefepime	31.0	27.8	26.6	26.4	25.8
Ceftazidime	24.3	23.6	22.3	20.5	21.5
Ticarcillin/Clavulanate	9.0	6.9	6.7	9.4	10.9
Amoxicillin/Clavulanate	14.4	15.9	14.6	11.0	12.9
Tobramycin	7.7	8.2	7.0	6.6	26.4
Cefoxitin	13.5	16.0	14.0	14.2	14.1
Piperacillin/Tazobactam	7.1	8.1	11.3	9.6	11.6
Meropenem	0.7	2.6	1.5	3.8	4.8
Imipenem	2.1	4.4	3.4	3.9	4.5
Cefoperazone/Sulbactam	6.8	7.2	7.1	5.8	6.6
Amikacin	2.1	2.9	3.5	1.9	5.8
Colistin	0.7	1.2	1.7	0.0	1.3
Ertapenem	3.2	5.9	4.5	4.3	4.8
Tigecycline	0.0	0.0	0.0	0.0	0.2

**Table 6:** Annual resistant proportion of *Klebsiella pneumoniae* from 2021 to 2025.

Antibiotic	2021 (n = 853)	2022 (n = 877)	2023 (n = 1059)	2024 (n = 832)	2025 (n = 746)
Cefuroxime	50.6	55.0	52.1	49.8	46.1
Ceftriaxone	47.9	53.4	51.5	45.7	43.0
Trimethoprim/Sulfamethoxazole	45.3	50.3	47.6	38.6	36.7
Ciprofloxacin	60.8	57.9	51.4	54.2	39.1
Levofloxacin	42.2	49.7	44.7	40.6	36.9
Aztreonam	58.3	52.6	48.2	48.9	36.6
Cefepime	43.5	47.2	43.8	41.9	36.7
Ceftazidime	39.2	46.0	40.7	38.6	31.9
Ticarcillin/Clavulanate	48.2	41.0	39.2	45.1	32.4
Amoxicillin/Clavulanate	30.7	37.3	33.7	30.6	32.0
Tobramycin	30.4	32.2	31.1	31.7	27.5
Cefoxitin	27.7	35.3	31.2	31.5	26.1
Piperacillin/Tazobactam	30.2	34.4	38.5	36.1	32.0
Meropenem	29.0	26.3	29.5	25.6	21.6
Imipenem	20.0	26.9	26.1	24.8	18.5
Cefoperazone/Sulbactam	27.2	33.5	29.6	27.6	22.3
Amikacin	19.5	26.6	21.8	21.0	16.4
Colistin	1.1	0.7	0.0	0.0	0.0
Ertapenem	20.2	28.3	25.5	23.7	18.9
Tigecycline	0.4	0.2	0.3	0.2	6.2

**Table 7:** Annual resistant proportion of *Pseudomonas aeruginosa* from 2021 to 2025.

Antibiotic	2021 (n = 563)	2022 (n = 518)	2023 (n = 662)	2024 (n = 592)	2025 (n = 603)
Ciprofloxacin	27.4	26.6	22.7	20.3	17.4
Levofloxacin	36.1	37.1	31.6	27.7	25.0
Cefepime	6.6	11.2	7.6	7.8	9.5
Ceftazidime	18.7	25.1	19.3	15.0	17.1
Ticarcillin/Clavulanate	33.6	41.7	35.8	29.9	31.0
Tobramycin	10.7	14.1	10.6	8.6	11.9
Piperacillin/Tazobactam	19.5	34.4	27.6	21.6	27.0
Meropenem	21.0	26.8	21.0	17.6	19.4
Imipenem	23.1	27.0	24.6	21.3	23.1
Cefoperazone/Sulbactam	19.0	27.2	20.2	15.4	20.2
Amikacin	4.6	5.2	5.7	5.1	8.0
Colistin	2.5	3.7	3.2	1.9	4.3

**Table 8:** Annual resistant proportion of *Acinetobacter baumannii* from 2021 to 2025.

Antibiotic	2021 (n = 410)	2022 (n = 339)	2023 (n = 454)	2024 (n = 334)	2025 (n = 264)
Trimethoprim/Sulfamethoxazole	44.4	52.8	45.2	47.0	49.6
Ciprofloxacin	61.5	62.2	59.0	65.0	58.3
Levofloxacin	55.1	56.0	53.5	59.6	53.0
Cefepime	53.7	49.3	51.1	55.7	56.4
Ceftazidime	60.2	62.2	57.7	65.3	60.6
Ticarcillin/Clavulanate	61.5	63.7	57.9	64.1	59.5
Tobramycin	52.9	56.0	53.7	50.6	52.7
Piperacillin/Tazobactam	62.7	64.3	58.8	67.4	61.7
Meropenem	60.7	62.8	58.1	65.9	61.7
Imipenem	61.5	61.4	57.0	65.0	60.2
Cefoperazone/Sulbactam	54.9	52.2	48.5	45.5	35.6
Colistin	0.2	0.6	0.4	0.0	1.9
Tigecycline	1.2	1.8	1.8	1.2	1.5

*E. coli* demonstrated high resistance to fluoroquinolones (ciprofloxacin >57%), trimethoprim/sulfamethoxazole (>47%), and broad-spectrum cephalosporins (cefuroxime, ceftriaxone >50%). A concerning upward trend was observed in resistance to aztreonam, rising from 24.6% to 34.7%. Resistance to tobramycin showed a notable increase in 2025 (26.4%, compared to 6.6% in 2024). Although still relatively low, resistance to carbapenems (meropenem, imipenem) and amikacin displayed an increasing trend over the five-year period, as shown in **Table 5**.

*K. pneumoniae* showed a declining trend in resistance to fluoroquinolones, cephalosporins, and carbapenems. For example, resistance to meropenem decreased from 29.0% to 21.6%. However, resistance to tigecycline increased (6.2%), as shown in **Table 6**.

*P. aeruginosa* exhibited an overall declining trend in resistance to most antimicrobial agents, particularly fluoroquinolones (ciprofloxacin decreased to 17.4%) and carbapenems (meropenem decreased to 19.4%). Low resistance rates were maintained for amikacin (≤8.0%) and colistin (≤4.3%), as shown in **Table 7**.

*A. baumannii* demonstrated high resistance to nearly all classes of antibiotics, including carbapenems (>57%), cephalosporins, fluoroquinolones, and piperacillin/tazobactam. Colistin and tigecycline were the only agents that maintained consistently low resistance proportion (≤1.9% and ≤1.8%, respectively), as shown in **Table 8**.

### Comparative analysis of trends in common resistant pathogens against CARSS surveillance data from 2021 to 2025

**Figure 2:**
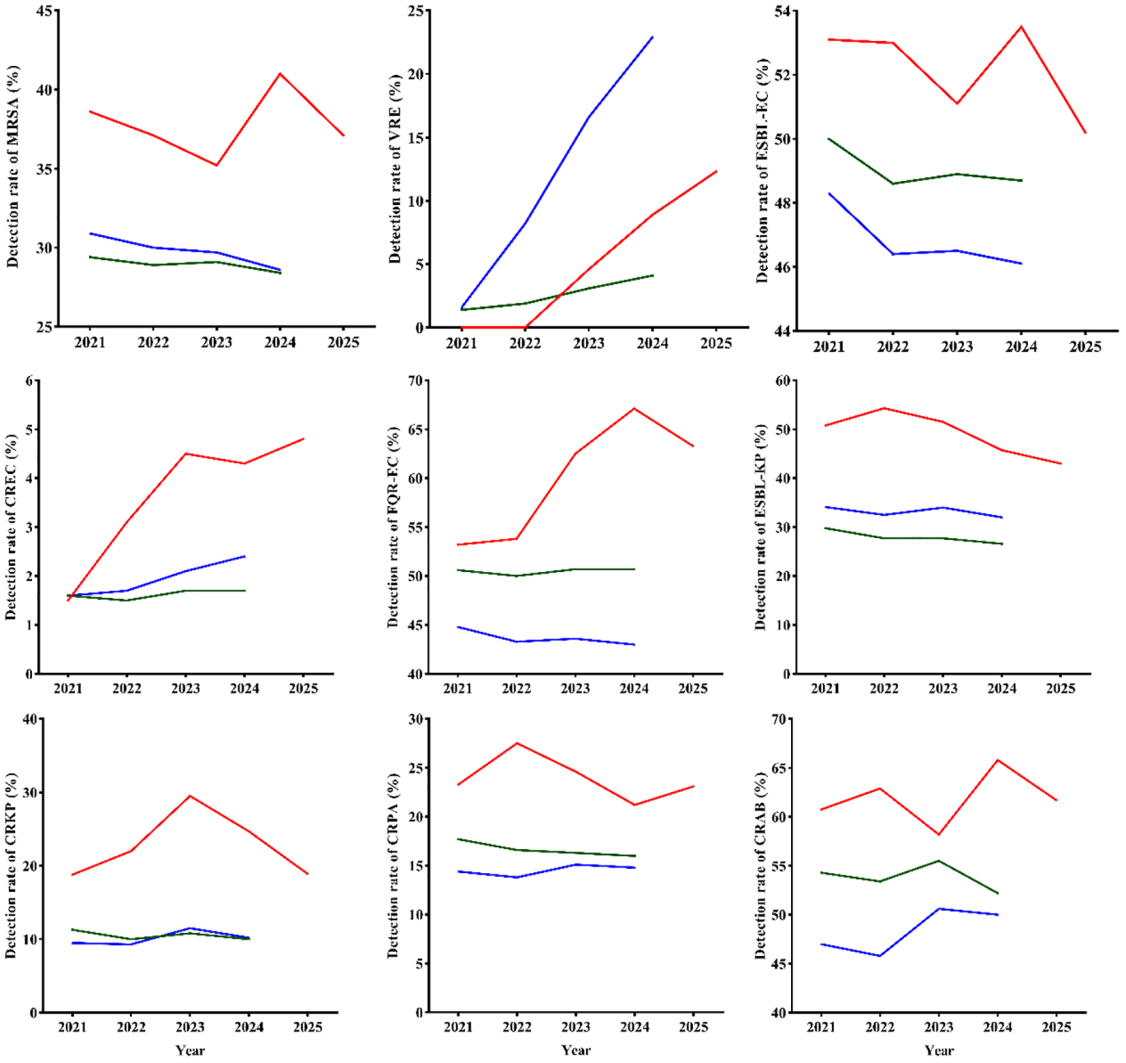
Comparative analysis of trends in common resistant pathogens against CARSS surveillance data from 2021 to 2025. MRSA, methicillin-resistant *Staphylococcus aureus*; VRE, vancomycin-resistant Enterococci; VREfm, vancomycin-resistant *Enterococcus faecium*; VREfs, vancomycin-resistant *Enterococcus faecalis*; ESBL-EC, extended-spectrum *β*-lactamase-producing *Escherichia coli*; CREC, carbapenem-resistant *Escherichia coli*; FQR-EC, fluoroquinolone-resistant *Escherichia coli*; ESBL-KP, extended-spectrum *β*-lactamase-producing *Klebsiella pneumoniae*; CRKP, carbapenem-resistant *Klebsiella pneumoniae*; CRPA, carbapenem-resistant *Pseudomonas aeruginosa*; CRAB, carbapenem-resistant *Acinetobacter baumannii*; CARSS, China antimicrobial resistance surveillance system. Red represents the detection rate of The Affiliated Guangdong Second Provincial General Hospital of Jinan University, blue represents the detection rate of Guangdong Province, China, and green represents the national detection rate of China.

**Figure 2** presents a comparative analysis of trends in common resistant pathogens against CARSS surveillance data from 2021 to 2025. The overall burden of MDROs at our hospital is significantly higher than the national and Guangdong provincial averages. This is particularly evident in CREC, CRKP, CRAB, and MRSA.

Notably, the detection rate of VRE has exhibited an outbreak-like increase since 2023. The carbapenem resistance rate in *K. pneumoniae* reached a peak of nearly 30% at our hospital and remains around 20%, markedly higher than the national (approximately 10%) and Guangdong provincial (approximately 10%) averages.

Similarly, the carbapenem resistance rate in *E. coli* has risen continuously to 4.8%, nearly triple the national level and double that of Guangdong. CRAB has maintained a persistently high resistance rate exceeding 60% at our hospital.

Additionally, quinolone resistance in *E. coli* is particularly pronounced, with the resistance rate surpassing 60% at our institution.

### Antimicrobial consumption and HAIs MDROs from 2021 to 2025

**Figure 3:**
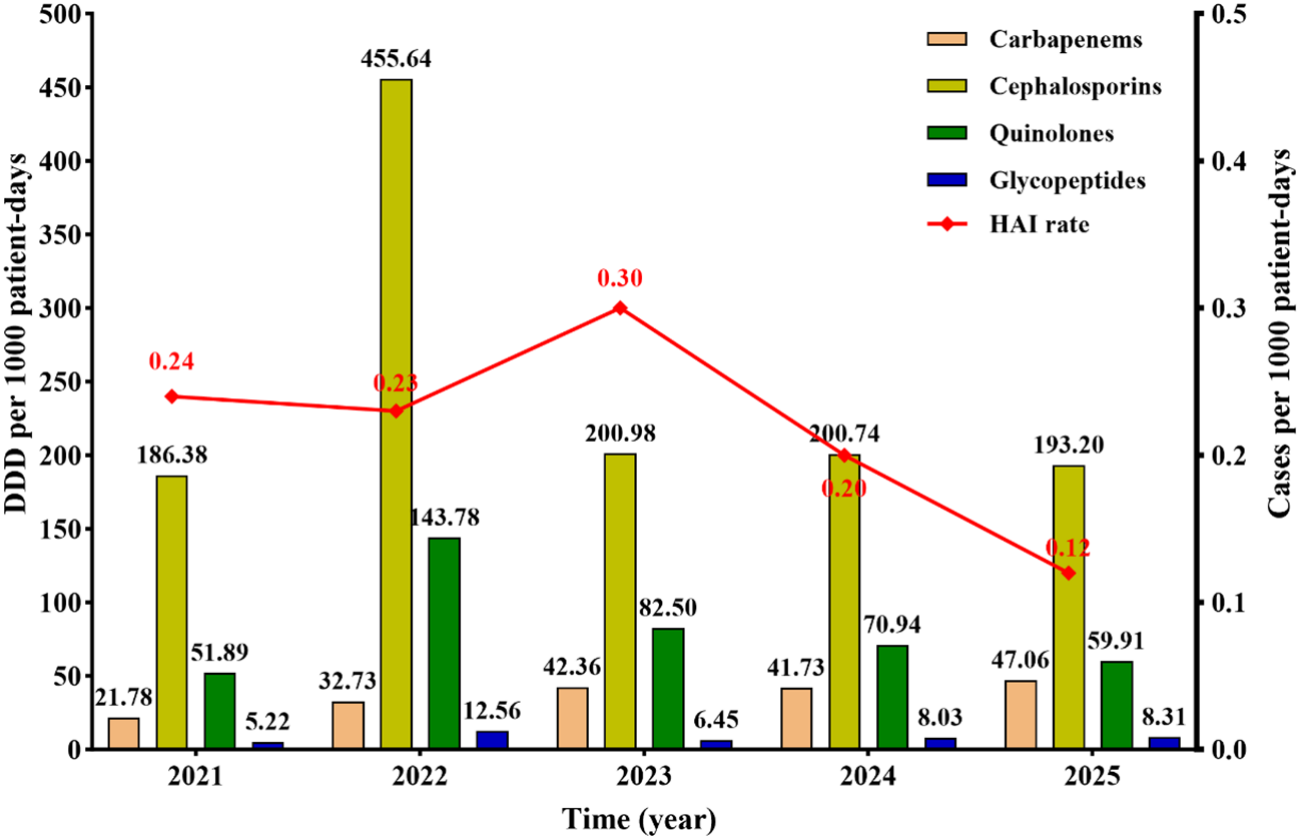
Trends in antimicrobial consumption and healthcare-associated MDROs infections from 2021 to 2025. DDD, Defined daily dose; HAIs, Healthcare-associated infections; MDROs, Multidrug-resistant organisms. The incidence of healthcare-associated MDROs infections, including MRSA, CREC, CRKP, CRPA, CRAB, and VRE, was expressed as cases per 1,000 patient-days.

**Figure 3** illustrates antimicrobial consumption and healthcare-associated MDROs infections from 2021 to 2025. The defined daily dose (DDD) per 1000 patient-days varied across antimicrobial classes from 2021 to 2025. Cephalosporin use peaked in 2022 at 455.64 DDD per 1000 patient-days, significantly higher than in other years, while carbapenem use demonstrated a sustained upward trend over the period. Quinolone consumption reached its highest level in 2022 (143.78 DDD per 1000 patient-days), declined in subsequent years, ultimately exceeding its 2021 baseline. Glycopeptide use rose to 12.56 DDD per 1000 patient-days in 2024, decreased, and then increased again, showing notable fluctuation in recent years. The HAIs incidence demonstrated dynamic changes during the 2021–2025 surveillance period. It rose from 0.24 cases per 1000 patient-days in 2021 to 0.30 cases per 1000 patient-days in 2023, followed by a notable decline to 0.12 cases per 1000 patient-days by 2025. A statistically significant reduction in the incidence of healthcare-associated MDROs infections was observed, from 0.256 cases per 1,000 patient-days in 2021–2023 to 0.160 cases per 1,000 patient-days in 2024–2025 (*p* < 0.001).

## Discussion

This five-year retrospective study provides a comprehensive analysis of pathogen distribution and AMR trends in a tertiary hospital in South China from 2021 to 2025. Consistent with previous reports (Qin et al., 2024; Liu Z. et al., 2025), our findings confirm that Gram-negative bacteria, particularly *E. coli*, *K. pneumoniae*, *P. aeruginosa*, and *A. baumannii*, persistently dominated the etiological landscape. Notably, while declining resistance trends were observed for several antimicrobial-pathogen combinations, such as carbapenems in *K. pneumoniae* and ESBL-KP, the overall burden of MDROs at our institution remained substantially higher than both national and Guangdong provincial averages, underscoring a persistent regional challenge. In response, enhanced infection prevention and control (IPC) measures targeting MDROs were implemented in 2024, which have effectively reduced the risk of nosocomial transmission of these pathogens.

Among Gram-negative pathogens, *E. coli* exhibited persistently high resistance to fluoroquinolones and third-generation cephalosporins (>50%), a finding consistent with observations from tertiary hospitals in Central and East China (Shi and Xie, 2023; Liu L. et al., 2025). Of particular concern was the steady increase in resistance to aztreonam (from 24.6% to 34.7%) and the rise in tobramycin resistance in 2025 (26.4%). Although carbapenem resistance remained relatively low, these antibiotics still retain utility for treating serious *E. coli* infections in the local setting. However, a continuous upward trend in resistance was observed over the five-year period, with annual resistant proportion now slightly exceeding the national average. This discrepancy may reflect regional pressures related to prescribing practices or pathogen transmission (Hu et al., 2022; Qin et al., 2024). Of particular concern, CREC ST410 has recently emerged as a major global health threat (Pitout et al., 2024), with surveillance data from Chinese hospitals (2017–2021) identifying ST410 as the most prevalent sequence type (Ba et al., 2024).

Other Gram-negative pathogens, including *K. pneumoniae* and *P. aeruginosa*, demonstrated encouraging declines in resistance to key broad-spectrum agents such as carbapenems and fluoroquinolones. For instance, meropenem resistance in *K. pneumoniae* fell from 28.9% in 2021 to 21.6% in 2025. A. baumannii proved to be the most formidable pathogen, as carbapenem resistance remained above 60% throughout the study period. The threat is further underscored by national data showing rates exceeding 70%, and reaching as high as 90% in some centers, highlighting the critical challenge posed by CRAB (Qin et al., 2024). CRAB transmission is most concentrated in high-intensity tertiary hospitals (Zheng et al., 2026). While expanded healthcare access is undoubtedly beneficial, it may inadvertently exacerbate nosocomial transmission and antimicrobial selective pressure if not matched by robust IPC and antimicrobial stewardship programs (Laxminarayan et al., 2013; Sun Jin and Fisher, 2021). In response, our institution implemented enhanced IPC measures targeting MDROs in 2024, which have since effectively reduced the risk of nosocomial transmission of these pathogens. *A. baumannii* strains in this study maintained favorable *in vitro* susceptibility to both tigecycline and colistin, suggesting that these agents may serve as cornerstone drugs for managing such multidrug-resistant infections (Yi et al., 2025).

Among Gram-positive pathogens, *S. aureus* remained the predominant species, with a notable surge in isolates observed from 2023 onward. Over the five-year period, MRSA, defined by oxacillin resistance, exhibited a fluctuating yet relatively stable trend, ranging from 35% to 41%. As we previously reported (Li et al., 2026b), resistance to penicillin G was nearly universal among MRSA strains in our hospital. MRSA has been documented to develop resistance to most antibiotics commonly used for its treatment, which severely limits therapeutic options. Inadequate infection prevention and control (IPC) measures, together with the ongoing indiscriminate use of antibiotics in both humans and animals, have facilitated the acquisition and spread of MRSA (Lakhundi and Zhang, 2018). We have also reported (Li et al., 2026b) that resistance to last-line agents such as vancomycin and linezolid remained negligible (<0.3%) in our setting, confirming their continued effectiveness against invasive MRSA infections.

Carbapenem use demonstrated a sustained upward trend over the study period, rising from 21.78 DDD per 1000 patient-days in 2021 to 47.06 DDD per 1000 patient-days by 2025. The decline in carbapenem resistance in *K. pneumoniae* and *P. aeruginosa* occurred despite increasing carbapenem use, suggesting that other factors (e.g., IPC measures) may have played a more dominant role in resistance trends for these pathogens (Wong et al., 2022).

A statistically significant reduction in the incidence of healthcare-associated MDROs infections was observed, from 0.256 cases per 1,000 patient-days in 2021–2023 to 0.160 cases per 1,000 patient-days in 2024–2025 (*p* < 0.001). This decline coincided with the implementation of intensified IPC measures in 2024, including weekly active surveillance for MDROs, improved contact isolation order compliance, as described in our earlier work (Li et al., 2026a). These findings provide strong evidence that active surveillance and strict contact isolation can effectively reduce healthcare-associated MDROs infections, even in the face of increasing antimicrobial consumption.

## Limitations

This study has several limitations. First, as a single-center retrospective analysis, our findings may not be generalizable to other regions or hospital types. Multi-center studies are needed to validate these observations. Second, the lack of molecular typing and resistance gene characterization limits our ability to confirm clonal transmission or identify specific resistance mechanisms underlying the observed trends. Third, antimicrobial consumption data were analyzed at the aggregate level rather than linked to individual patients, precluding patient-level correlation between antibiotic exposure and resistance development. Four, due to the retrospective design and limitations of our data collection system, we were unable to adjust for potential confounding factors such as patient case mix, severity of illness, and prior hospitalization history. Consequently, we cannot rule out the possibility that the observed AMR burden may be influenced by the distribution of clinical conditions within the study population. For example, a higher proportion of patients with multiple or recurrent infections may reflect greater antimicrobial exposure, which could in turn promote the emergence and selection of resistant organisms. Future prospective studies incorporating detailed clinical data are needed to address these confounding effects. Five, Future studies should further investigate the distribution of MDROs by specimen type to identify which specimen sources are significantly associated with MDROs isolation, as this would help guide diagnostic priorities, infection prevention efforts, and targeted antimicrobial stewardship interventions. Finally, The small sample size (n=5) limits the statistical power and robustness of regression-based trend analyses, and the results should be interpreted as exploratory rather than confirmatory. Longer term studies with more annual data points are needed to validate the observed trends. Due to limitations of our data collection system, we were unable to retrospectively retrieve the specific resistance profiles (e.g., carbapenem-resistant, cephalosporins-resistant, quinolone-resistant, or glycopeptide-resistant backgrounds) for each individual healthcare-associated MDROs infection event. Consequently, we could not stratify HAI-MDROs rates by resistance background. Future prospective studies should collect detailed resistance profiles to enable such stratified analyses.

## Conclusion

In conclusion, this five-year study reveals a persistently high burden of MDROs in a tertiary hospital in South China, particularly among Gram-negative pathogens. Our findings demonstrate that intensified IPC measures, including active surveillance and improved contact isolation compliance, can effectively reduce healthcare-associated MDROs infections, even against a backdrop of increasing antimicrobial consumption. These results underscore the equal importance of antimicrobial stewardship and infection control in combating antimicrobial-resistant organisms and provide evidence-based support for hospital policies aimed at MDROs prevention and control.

## Data Availability

The original contributions presented in the study are included in the article/supplementary material. Further inquiries can be directed to the corresponding author.
